# Overcoming Drug Resistance and Treating Advanced Prostate Cancer

**DOI:** 10.2174/138945012802429615

**Published:** 2012-09

**Authors:** Julius Semenas, Cinzia Allegrucci, Stephen A Boorjian, Nigel P Mongan, Jenny Liao Persson

**Affiliations:** 1 Division of Experimental Cancer Research, Department of Laboratory Medicine, Lund University, Clinical Research Centre in Malmö; 2 Faculty of Medicine and Health Sciences, School of Veterinary Medicine and Science, University of Nottingham, LE12 5RD, United Kingdom; 3 Department of Urology, Mayo Clinic, Rochester, MN, USA

**Keywords:** Bone nietasrasis, novel therapy, prostate cancer, treatment resistance.

## Abstract

Most of the prostate cancers (PCa) in advanced stage will progress to castration-resistant prostate cancer (CRPC). Within CRPC group, 50-70% of the patients will develop bone metastasis in axial and other regions of the skeleton. Once PCa cells spread to the bone, currently, no treatment regimens are available to eradicate the metastasis, and cancer-related death becomes inevitable. In 2012, it is estimated that there will be 28,170 PCa deaths in the United States. Thus, PCa bone metastasis-associated clinical complications and treatment resistance pose major clinical challenges. In this review, we will present recent findings on the molecular and cellular pathways that are responsible for bone metastasis of PCa. We will address several novel mechanisms with a focus on the role of bone and bone marrow microenvironment in promoting PCa metastasis, and will further discuss why prostate cancer cells preferentially metastasize to the bone. Additionally, we will discuss novel roles of several key pathways, including angiogenesis and extracellular matrix remodeling in bone marrow and stem cell niches with their relationship to PCa bone metastasis and poor treatment response. We will evaluate how various chemotherapeutic drugs and radiation therapies may allow aggressive PCa cells to gain advantageous mutations leading to increased survival and rendering the cancer cells to become resistant to treatment. The novel concept relating several key survival and invasion signaling pathways to stem cell niches and treatment resistance will be reviewed. Lastly, we will provide an update of several recently developed novel drug candidates that target metastatic cancer microenvironments or niches, and discuss the advantages and significance provided by such therapeutic approaches in pursuit of overcoming drug resistance and treating advanced PCa.

## INTRODUCTION

### Increased Incidence of Prostate Cancer

Prostate cancer (PCa) develops in the prostate gland of male reproductive system. PCa has been recognised to be an androgen driven disease since the 1941 discovery by Huggins and Hughes [1]. In the intervening years much research has focussed on the determining the underlying pathogenic molecular mechanisms of androgens and the AR in PCa. Androgen receptor (AR)-mediated signaling play essential roles in PCa initiation and progression. Most primary prostate tumor cells are initially sensitive to androgen deprivation therapy (ADT) [2]. However despite the advances in PCa treatment, reduction in mortality rates, and increased patient survival, PCa still remains the most common non-cutaneous malignancy and the third leading cancer-related cause of death among men in developed countries. The most recent available reports from World Health Organisation (WHO) suggests that 75% of all new PCa cases are diagnosed in developed countries, with worldwide PCa prevalence accounting for approximately 899,000 new cases, and as many as 258,000 deaths each year [3]. In US alone, as many as 240,890 new cases, and 33,720 PCa-related deaths were estimated to occur in 2011, and account for almost 29% of all cancer cases, and 11% of all cancer-related deaths in US male population [4]. Most recent cancer estimates suggest that in 2012, the prevalence of PCa in the US will show a slight increase, totalling up to approximately 241,740 new cases [5]. Estimations of PCa prevalence and mortality for 2012 from other parts of the world are currently unavailable. Nevertheless, the available data suggests that PCa remains one of the major burdens in male population in the developed countries.

### The Causative and Risk Factors Associated with PCa

Well-established risk factors for prostate cancer include aging, ethnicity, and family history of the disease [6]. In addition to that, hormones, genetic susceptibility, obesity, tobacco and alcohol consumption, high burden of cancer-related infections or inflammations, sexually transmitted diseases, lack of exercise, and high consumption of animal fat or meat have also been suggested to predispose to PCa [7, 8]. American Cancer Society has reported that risk for developing PCa is one in 10,000 in men younger than 40 years of age, while it increased to one in seven in men over 60 years old. Moreover, African American ethnicity has been shown to also predispose to the risk [9]. Various studies have reported that aging-induced changes in gene expression are related to PCa development. In 2005 Begley and colleagues showed that CXCL12 overexpression and secretion by aging fibroblasts promotes the growth of human prostate epithelial cells [10]. Aging-related changes in the prostate microenvironment also appear to be a potential contributor to the progression of prostate neoplasia [11]. While the various risks of developing PCa are being studied, PCa preventive studies aiming to identify potential factors that reduce PCa occurrence have also been reported. However, these studies investigating anti-aging supplement and certain compounds such as Vitamin E and selenium have not shown significant PCa-preventive effects [12]. PCa incidence has increased over recent decades. It is likely that environmental factors which may predispose to PCa combined with an aging western population contribute to the overall PCa incidence. However the single most prominent factor likely leading to the observed increased incidence of PCa is the development of diagnostic screening for serum levels of prostate-specific antigen (PSA) [13]. 

### Transition of Localized PCa to Metastatic Disease

Statistical data suggests that PCa is generally a slowly progressing disease [14, 15]. Despite our increasing understanding of the causes of PCa, the cellular and molecular mechanisms which enable localized PCa to invade and metastasize remain poorly understood. Moreover, it remains unknown how long it takes for an organ-confined primary tumor to develop into a highly invasive PCa [6]. Indeed, it is not yet possible to diagnostically distinguish indolent localized prostate tumors, which possess little metastatic potential, from aggressive localized prostate tumors with high metastatic potential. Nevertheless, biochemical recurrence (BCR), defined by increased serum PSA levels following prostatectomy or radiation therapy for clinically localized PCa, has been shown to predict metastatic progression (MP) and prostate cancer-specific mortality (PCSM) by a median of 8 years and 13 years, respectively [16, 17]. This suggests that it may take 8 to 13 years for a primary PCa to progress towards lethal metastatic disease. As we will discuss later, no curative therapy exists for metastatic PCa. 

It is now established that bone is the most common preferential site of PCa metastasis. A study by Coleman *et al*. have reported that in post-mortem examinations, approximately 70% of patients who have died from PCa complications show evidence of metastatic bone disease [18] with common site for bone metastasis being in the axial skeleton (skull, vertebra, ribs and collar bone, scapula, and proximal femur) [19]. Since bone is the most common site for PCa metastasis it is crucial to understand the underlying mechanisms that facilitate this preferential migration of circulating PCa cells to the bone. There is now compelling evidence which suggests that disseminated tumor cells (DTCs) migrate to the bone marrow using mechanisms similar to those that are commonly exploited by homing hematopoietic stem cells (HSCs) during bone marrow transplantation. Bone cells and endothelial cells that form the bone marrow stem cell niches express several key chemokine receptors. Similarly, cancer cells also express high levels of the chemokine receptors which will then preferentially interact with the bone marrow niches. Muller and colleagues have shown that chemokine receptors CXCR4 and CXCR7 are highly expressed in human breast cancer specimens, while their corresponding ligands CXCL12/SDF-1alpha (stromal cell-derived factor) and CCL21/6Ckine showed peak levels in primary breast cancer destination sites [20]. Similarly, Taichman and colleagues have confirmed the importance of SDF-1/CXCR4 in both *in* *vivo* and *in vitro* PCa models in [21]. Other studies have also reported similar results [22-25]. Furthermore, the Annexin II receptor expressed on osteoblasts and endothelial cells in HSC niche has also been shown to regulate cell adhesion, migration, homing, and growth of prostate cancer [26]. Thus the chemokine rich milieu of the bone marrow combined with adhesion molecules expressed within the HSC niche are believed to be crucial in recruiting DTCs promoting PCa metastasis in the bone Fig. (**1**).

### Detection and Diagnosis of PCa Bone Metastasis

During PCa progression to metastases, patients may experience various symptoms that are associated with PCa. These include but are not limited to various urinary problems, and persistent pain in the lower back, hip, or upper thigh regions [6]. Nevertheless, statistical data suggest that the large majority of incident PCa cases are asymptomatic, and may only be detected through screening [14, 15]. 

PCa is in fact most commonly diagnosed through screening, which may be performed through digital rectal examination (DRE), and/or blood tests for prostate-specific antigen (PSA). As the majority of all prostate carcinomas arise in the peripheral zone of the prostate, DRE may be effectively used to detect tumerous growths on the periphery of the gland. Limitations of DRE include limited sensitivity and reduced number of detections as compared to PSA blood test. In this context, the number PCa diagnosis has increased significantly concurrently with the development of the PSA serum immunoassay in 1980 [13, 27]. The use of DRE and PSA screening is now a standard procedure in some countries, and has been typically performed annually on asymptomatic men beginning at 50 years of age. Depending on the examination results, additional procedures like transrectal ultrasound or transrectal ultrasound-guided biopsy may be performed, and as such may be used to assess the potential presence of PCa [6].

Where there is potential metastatic involvement, the most common methods used to detect bone metastasis involve ^99m^Tc-methylene diphosphonate (MDP) bone scintigraphy (BS) and X-ray radiography [28]. Apart from ^99m^Tc-MDP, an increased level of serum alkaline phosphatase, which is a marker for osteoblastic proliferation, has also been associated with PCa metastasis [29]. New markers such as ^11^C- or ^18^F-labeled choline and acetate, ^11^C-methionine, and ^18^F-fluorodyhodrotestosterone are currently under investigation, with the aim of replacing ^99m^Tc-MDP in diagnostics [30-33]. As such, metabolic imaging by positron emission tomography (PET) and magnetic resonance imaging (MRI) are rapidly gaining interest. In this context, MRI of the skeleton has been shown to be a highly sensitive method of detecting bone metastasis [28], and to be a reliable tool to quantify PCa metastasis, and to measure tumor response to therapy [34], while advantages of PET over MRI are yet to be demonstrated [35]. 

### Treatment and Treatment Prediction of Various Stages of PCa

Upon PCa diagnosis, conventional therapeutic approaches to treat PCa include active surveillance, surgery, radiation therapy, hormone therapy, and chemotherapy. A combination of these therapies may also be applied. The decision on which therapeutic approach is applied is based on number of factors primarily involving clinical and histopathological stage and grade of the tumor, and patient’s age and general health. Active surveillance is suggested if the side effects of the treatment are thought to be greater than the potential benefits (specifically for patients with a < 10 year estimated life expectancy), or if patient is diagnosed with an early, or slow-growing PCa [6]. Upon diagnosis of stage I-II PCa (and conditionally upon diagnosis of stage III-IV PCa), surgery may be applied to remove the prostate (prostatectomy). Recent developments in robotic-assisted laparoscopic prostatectomy procedures have offered clinical advantages over conventional open surgical approaches, though evaluation of oncological outcome is awaited [36].

Since PCa is initially dependent upon AR signaling, hormone therapy (also referred to as androgen deprivation therapy or ADT) is often used to target and reduce AR-mediated growth of the tumor [37, 38]. This may be achieved either through surgical or chemical castration. Surgical castration involves the removal of testicles (orchiectomy) with the effect of reducing testosterone biosynthesis. However as adrenal and local intra-tumor androgen biosynthesis is unaffected, sufficient androgens may still be available to promote PCa proliferation. For this reason surgical castration may be used in combination with AR blockade to achieve total androgen blockage. Chemical castration refers to use of various pharmaceuticals such as, luteinizing hormone-releasing hormone (LH-RH) agonists, anti-androgens, or other testosterone synthesis-inhibiting drugs, which reduce androgen levels or block AR function. A combination therapy of the two ADT may be used to achieve total androgen blockage (also known as combined androgen blockage). Alternatively, radiotherapy may be applied at any given stage of PCa, and may involve external beam radiotherapy (EBRT) as well as internal (brachytherapy) irradiation. Hormone therapy is commonly applied in combination with radiotherapy (before, during and after), and may also be applied as a standalone therapy for recurrent PCa. However, a significant advantage of hormone monotherapy is yet to be demonstrated [6]. 

PCa progression and metastasis in recurrent PCa are common, and the rate of prostate cancer-specific mortality remains high [5]. Once PCa metastatic progression has been confirmed, ADT is used as the primary therapy and has been shown to be responsive in as many as 80% of metastatic PCa cases [39, 40]. However, statistical data has shown that use of ADT only marginally improves the OS [41], and disease typically progresses into a castration resistant prostate cancer (CRPC) [42, 43]. Interestingly, although CRPC are no longer dependent on androgen stimulation, AR-mediated growth promoting pathways remain important therapeutic targets in CRPC [44-46]. Overall median survival with metastatic disease has been estimated to be approximately 30 months, with only 10% of patients living 10 years beyond diagnosis [39, 40, 47]. Currently, patients with metastatic CRPC are treated with taxane-based chemotherapy with such chemotherapeutic drugs as docetaxel and cabazitaxel in combination with predisone [48-50]. The two therapies have been approved after two phase III clinical trials which concluded superior benefit of docetaxel or docetaxel in combination with prednisone as compared to other treatments [49, 50]. However, docetaxel therapies have shown only modest improvement in OS (of approximately 3 months) when compared to miloxantrone and prednisone treatment groups [51]. 

## DRUG RESISTANCE

Drug resistance refers to the status of poor responsiveness of tumor cells to chemotherapeutic drugs. Multiple mechanisms of drug resistance in cancer have been described, but typically involve efflux mechanisms and membrane associated changes to prevent drug accumulation within tumor, the acquisition of mutations which block drug action at the target, and cellular adaptation to drug treatment whereby the drug targeted pathway is bypassed. Thus aggressive cancer cells often gain new and advantageous mutations leading to increased survival, and therefore cannot be eradicated by apoptosis-inducing agents. For example, there are several intrinsic mechanisms utilized by tumor cells to escape drug selection or increase survival upon the induction of the therapeutic agents. These include activation of AR signaling networks that regulate PCa cell proliferation, and EGFR, VEGFR signal transduction pathways [52]. In addition there is evidence that multiple pathways converge to mediate drug resistance in CRPC, including activation of Akt/PI3K and MAPK/ERK [53, 54], rapamycin (mTOR) [55], nuclear factor-kappa B (NFκB)/IL-6 [56], Hedgehog [57], endothelin A receptor (E_A_R) [58], and somatostatin receptor [59] signaling pathways. As we will outline below there is evidence that PCa drug resistance may arise within PCa cells exploiting structures within the tumor micro-environment or stem cell niches to acquire invasive and survival advantages Fig. (**2**). Moreover, current cytotoxic therapies fail to target quiescent cancer stem cells as summarized by Visvader JE and Lindeman GJ [60]. 

### Intrinsic AR-Associated Mechanisms of Drug Resistance

It has been well established that activation of AR by androgens stimulates proliferation and inhibits apoptosis of PCa cells. In the absence of androgens, the AR is believed to reside in an inactive form in the cytoplasm bound to heat-shock proteins such as HSP90. Once testosterone enters the cell it can be converted to a more potent derivative, dihydro-testosterone (DHT) by 5-α reductase. Both testosterone and DHT can bind AR and induce a conformational change that leads to dissociation of AR from the heat-shock protein, AR phosphorylation, and homo-dimerization. These events consequently lead to translocation of AR into the nucleus, where it can exert its effects as a transcription factor Fig. (**1**). The AR is a member of the nuclear receptor superfamily of ligand dependent transcription factors [61]. In the presence of testosterone or DHT, the AR recruits multiple transcriptional coregulator complexes harbouring distinct enzymatic activities, including histone acetylation, methylation and demethylation functions which cooperate to enable transcriptional activation to occur. Aberrant expression of AR coregulators have been implicated in prostate cancer, most notably the lysine specific demethylase-1 (LSD1) [62, 63] which appears to possess important functions in hormone dependent and CRPC [64] Fig. (**3**). 

During the transition from hormone dependent PCa to castration resistant disease, it has been shown that, despite continued maintenance of circulating castrate testosterone levels and the presence of truly androgen-independent tumor clones, the majority of PCa cells remain dependent on AR-mediated signaling [65, 66]. It has been suggested that intrinsic-mechanism mediated either through increased AR expression, amplification of the AR gene, or mutation-induced changes hormone responsiveness of the AR [67-69] contribute to ADT resistance. In addition, other mechanisms of AR signaling maintenance have also been reported. Stanbrough and colleagues compared gene expression profiles of androgen-independent PCa bone marrow metastases (N=33) with primary PCa samples (N=22) and reported that while AR expression was upregulated in metastatic samples, additional genes including the aldo-keto reductase family 1 member C3 (AKR1C3), a key prostatic enzyme that reduces adrenal androstenedione to testosterone, was also overexpressed [70]. This suggests that despite ADT, androgens are continuously produced within the tumor. Montgomery and colleagues further reported that testosterone levels within metastatic PCa samples were increased compared to primary PCa tumor specimens from anorchid men, reflecting increased expression of steroidogenic enzymes within the metastatic PCa lesion [71]. Collectively these studies have shown that while ADT decreases serum testosterone levels by approximately 95%, intra-tumor androgen levels combined with overexpressed AR may be sufficient to drive the progression of PCa in AR-dependent manner. In addition to that, findings of transactivation of AR in presence of 5α-androstane-3α, 17β-diol (androstanediol, which can be converted into DHT) were also recently reported [72]. Intra-tumor and serum androgen levels (testosterone and DHT) in recurrent PCa appear to be sufficient to activate AR, and suggested that the PCa microenvironment may be capable of intracrine androgen biosynthesis [73]. Furthermore distinct AR signaling networks operate in hormone dependent PCa and CRPC [44]. A recent study has shown that androgen levels in CRPC are sufficient to increase AR expression and activate AR-signaling pathways which support hormone independent cellular proliferation [64]. Thus ADT strategies which systemically reduce androgen levels may accelerate the transition of hormone dependent PCa towards a CRPC state. 

Furthermore, there is now evidence indicating that taxane-based chemotherapies act in CRPC in part by blocking β-tubulin mediated AR-nuclear translocation. Thus mutations in β-tubulin related to taxane resistance further contribute to animportant role for the AR in CRPC [74]. Various additional taxane resistance mechanisms have been described including upregulation of membrane-bound efflux proteins such as ATP-binding cassette (ABC) transporters P-glycoprotein (P-gp)/MDR1 (ABCB1), MDR2 (ABCB4), or MRP1 (ABCC1) [75], direct mutation-driven alterations of the drug target such as tubulin [76] or defects in apoptotic pathways of the target cells [77].

### Intrinsic Proliferation- and Survival Pathways -Mediated Drug Resistance

Increasing evidence suggest activation of alternative survival pathways in CRPC. Seruga and colleagues have summarized drug resistance mechanisms in PCa, where they suggest that the alternatively-activated survival pathways may include activated receptor tyrosine kinases (RTKs) [78]. Moreover, epidermal growth factor (EGFR) and vascular endothelial growth factor receptor (VEGFR) are linked to signaling transduction pathways including Akt/PI3K or Ras/Raf/MEK/ERK pathways, which mediate cell proliferation and survival. Evidence for the involvement of these pathways in CRPC context has been extensively demonstrated by multiple studies. Treatment with various agents targeting these and other pathways such as mammalian target of mTOR [55], MAPK/ERK [53, 54], VEGF, and its receptor VEGFR, have also been reported to be regulated by androgens in androgen-dependent tumors through activation of HIF1α [79]. Androgen depletion leads to direct up-regulation of VEGF-C, which in turns activates AR coactivator BAG-1L expression that enhances AR transactivation [80]. Activation of other receptors and their pathways, such as interleukin 6 (IL-6) or Wnt/β-catenin has also been reported to be involved in the crosstalk with AR [81]. Similarly, Insulin-like growth factor 1 (IGF1) has also been reported to enhance AR function in low or absent androgen levels, and may promote the transition towards androgen-independence [82, 83]. Transforming growth factor β (TGFβ) was also reported to be overexpressed in PCa, and shown to exert diverse functions in stromal tumor cells *via* SMAD-dependent or SMAD-independent signaling pathways [84]. Nuclear factor-kappa B (NFκB)/IL-6 [56], Hedgehog [57], E_A_R [58], and somatostatin receptor [59], have shown to either enhance or completely restore sensitivity to taxane-based therapy. These findings suggest that alternative signaling pathways may play a central role in CRPC and drug resistance, and provide valuable insight of overcoming the resistance by targeting these pathways.

### Extrinsic Mechanisms of Drug Resistance

Tumor cell interaction with the surrounding microenvironment plays an important role in PCa bone metastasis. Indeed environment-mediated drug resistance (EMDR) arises whereby tumor cells are protected from various types of apoptosis-inducing agents through rapidly induced signaling events in their microenvironment rather than acquired resistance [85]. The evidence for such mechanisms comes from both *in vitro* and *in vivo* studies that demonstrated the presence of surviving tumor cells immediately after therapy. Teicher and colleagues have reported that EMT-6 murine mammary tumor cells survived following exposure to various types of chemotherapeutic drugs such as *cis*-diammine-dichloroplatinum (CDDP), carboplatin, cyclophosphamide (CTX), or thioTEPA *in vivo*, whereas no resistance was observed in the same cells exposed to the these drugs *in vitro* in the absence of tumor [86]. These findings suggest that microenvironment plays an important role in some aspects of drug resistance.

EMDR can be classed into two phenotypes: soluble factor-mediated drug resistance (SFM-DR) and cell adhesion-mediated drug resistance (CAM-DR), and was summarized by Meads an colleagues [85]. SFM-DR phenotype is induced by cytokines, chemokines and growth factors secreted by fibroblast-like tumor stroma, while CAM-DR is induced by adhesion of tumor cell integrins to stromal fibroblasts, or to extracellular matrix (ECM) proteins. Tumor cell integrins are able to bind to various secreted proteins on ECM, or receptors such as vascular cell adhesion protein 1 (VCAM1) expressed on stroma, which induce quiescence, modulate pro- and anti-apoptotic molecules, and consequently lead to CAM-DR. At the same time, paracrine amplification loop of soluble factors such as interleukin 6 (IL-6), fibroblast growth factor (FGF), vascular endothelial growth factor (VEGF), stromal cell-derived factor 1 (SDF1, also known as CXCL12), and others may lead to SFM-DR. Vaday and colleagues have developed fully human single chain Fv antibodies (scFv) against CXCR4, and studied the inhibitory effects of the said antibodies on CXCR4 in PC-3 and LNCaP cell line models. They have discovered that binding of scFv antibodies to CXCR4 on cell surface downregulated CXC12-induced calcium mobilization in the cells, and dramatically reduced PCa cell migration towards CXCL12, as well as invasion through ECM gel [87].

In the context of SFM-DR and alternative survival pathway activation in CRPC, chemokine (C-C motif) ligand 2 (CCL2; monocyte-chemoattractant protein-1) has been reported to be a prominent modulator of metastatic growth of PCa in bone [88]. Zhang *et al*. have demonstrated that overexpression of CCL2 by bone marrow osteoblasts, endothelial cells, stromal cells, as well as PCa cells leads to protection of PCa cells from death through autophagy, and is mediated through activation of protein kinase B or Akt/PI3K survival pathways. Showing that inhibition of CCL2 substantially decreased PCa growth, and associated aggressive phenotype in animal models, Zhang *et al*. proposed that targeting CCL2 in the tumor microenvironment may be an attractive therapeutic target in metastatic PCa. Indeed the role of CCL2 in drug resistance setting has been further demonstrated by Qian *et al*., who have reported that following chemotherapy, levels of CCL2 are increased, lead to activation of Akt/PI3K signaling pathway, and therefore may enable PCa to escape docetaxel induced cytotoxicity [89].

Because a number of anti-cancer drugs access solid tumors *via* the bloodstream, it may be difficult for the drugs to reach and penetrate a solid tumor at sufficient concentrations to exert their anti-cancer effects [90]. Growing tumors, just as any normal tissue, require a constant supply of oxygen and nutrients, and therefore release various angiogenic growth factors to promote angiogenesis and to increase blood supply. Nevertheless, tumor blood vessel networks are highly disorganized, and have lower than normal blood flow. Consequently, nutrient and oxygen delivery to the tumor sites including PCa is often impaired, rendering regions of PCa tumors relatively hypoxic [91]. For this reason poor blood supply may impair drug delivery of such agents as doxorubicin or taxanes [92, 93]. Drug delivery may also be impaired by increased interstitial fluid pressure within the tumor due to poorly formed lymphatic drainage [94]. Moreover, hypoxic conditions have also been described as favourable for survival and metastasis of various types of cancer, including PCa through activation of hypoxia-inducible factor such as HIF1α [95].

The two EMDR phenotypes CAM-DR and SFM-DR can be readily observed in *in vitro* models. However, it is likelythat both EMDR mechanisms cooperate* in vivo*. Evidences in support of this were reported in myeloma and small cell lung carcinoma (SCLC), where secreted factor, SDF1, has been shown to increase β1 integrin-mediated adhesion of myeloma [96] and SCLC [97] cells. Furthermore, SDF1 is also known to enhance integrin-mediated binding of tumor cells to ECM [98]. Tumor-stroma cooperativity has also been demonstrated to increase the CAM-DR phenotype through modulation of extracellular matrix (ECM) composition. For example expression of collagen IV was higher in bone marrow of patients with multiple myeloma as compared control patients [99]. Given the importance of CAM-DR and SFM-DR in orchestrating resistance, investigation of these pathways in bone metastatic PCa is warranted. 

### Extrinsic Mechanisms of Drug Resistance: Cancer Stem Cells

Small numbers of adult stem cells are believed to exist in most if not all adult tissues including the prostate gland [100, 101]. These cells play essential roles in tissue maintenance and repair. Although the exact identity of prostate stem cells remains controversial, the ability of the prostate gland to renew in animal models following successive rounds of androgen ablation and stimulation has been attributed to prostate stem cells. Putative prostate stem cells have been identified by virtue of expression of “stemness” associated markers [102-106].

Although the precise cellular origin of solid cancers remains controversial, increasing evidence indicates that tumors contain a reservoir of transformed stem cells or cancer stem cells (CSC) responsible for tumor initiation, progression and metastasis [101]. There are at least two potential origins of CSCs. According to the CSC hypothesis, transformation of normal tissue stem cells by progressive acquisition of genetic mutations and epigenetic changes generate a small population of CSC that retain the ability to self-renew and differentiate to the cellular lineages that comprise the heterogeneous bulk of the tumor [102-104, 106, 107]. Other studies show that stochastic events induced by the tumor microenvironment can enable tumor cells to adopt a malignant stem cell-like phenotype which function to repopulate tumor masses [108]. Such transformation events can also reprogram luminal prostate cells into cancer cells with CSC characteristics. This has been demonstrated for luminal progenitor cells expressing the homeobox gene NKx3.1 that can be targeted for transformation in castration–resistant disease. In the absence of the tumor suppressor gene *PTEN*, these cells behave like CSC and show bipotential differentiation potential [109]. The expression of homeobox genes, which normally regulate pluripotency and self-renewal in embryonic stem cells, is associated with drug resistance in PCa. Likewise, over-expression of the pluripotent marker NANOG confers CSC properties. Prostate CSC expressing the pseudogene *NANOGP8*, coding for a functional protein with *NANOG* activity, have been shown to exhibit enhanced cell clonal growth and tumorigenicity. Importantly, *NANOG* induction induces castration-resistance tumor development from LNCaP cells [110]. The fact that CSC share many features with normal tissue stem cells, including inherent resistance mechanisms, is not surprising. However, CSC can arise stochastically from selective pressure during tumor growth and evolve selective mechanisms that confer survival and growth advantage, such as the epigenetic reprogramming and expression of embryonic stem cell genes. It is for this reason that an accurate screening of drug resistant CSC, preferably expanded through serial xenografts, is necessary to understand the molecular pathways responsible for minimal residual disease. 

CSC share many characteristics with normal stem cells which limit the efficacy of current therapies, mainly due to their quiescent nature, but also because of expression of drug transporters and altered DNA damage response mechanisms. These findings are discussed in further detail in review by Visvader JE and Lindeman GJ [111]. Prostate CSC, like their normal counterparts, are AR negative and as such are unaffected by ADT [105]. Microarray analysis of CD133^+^/α2β1 integrin^high^/CD44^+^ human prostate CSC population identified the JAK-STAT, NF-κB and focal adhesion signaling pathways as key CSC molecular signature. The observation that elevated levels of *STAT1 *gene correlates with docetaxel resistance in PCa, suggests role for CSC in drug resistance [112]. CSC demonstrate multidrug resistance due to high expression of drug efflux and detoxifying enzymes, and different DNA damage response involving resistant to apoptosis combined with enhanced DNA repair mechanisms and quenching of reactive oxygen species [113]. Drug resistant CSC can therefore be accountable for residual disease and cancer recurrence. Consistent with this, human PCa cell lines resistant to docetaxel or mitoxantrone display an enriched CSC population which over-express ABCG2/BCRP and MDR1/Pgp transporters. Microarray analysis of taxane resistant CSC identified an enriched expression of the pluripotency stem cell gene *POU5F1/OCT4* in these cells, which is directly associated with their increased tumorigenicity [114]. 

While *de novo *drug resistance may be responsible for protecting tumor cells from initial therapies, it may also lead to surviving foci of residual disease and post-treatment recurrence. Such foci of surviving cells are therefore a subject to selective pressure and may lead to development of complex drug resistance mechanisms, and increased aggressiveness of the surviving cells [85]. Selection for CSC clones with survival advantages due to continuous chemotherapy treatment has been reviewed elsewhere [111, 115]. These CSC clones may in fact represent the small subpopulation of cells that after therapy are referred to as minimal residual disease (MRD), have multidrug resistance, and are capable of repopulating the tumor. Understanding CSC evolution during prostate tumor progression and following treatment is essential for the design of therapies that can specifically target tumor initiating CSCs. 

### Extrinsic Mechanisms of Drug Resistance: Stem Cell Bone Marrow Niche

As noted earlier, once PCa metastasize to the bone, there are no available curative therapies [19, 116, 117]. Therefore, it an urgent need remains to gain a better understanding of the mechanisms underlying PCa bone metastasis and drug resistance. Jung and colleagues recently analyzed 10 serum bone turnover markers in sera of 117 PCa patients with localized disease (n = 39), nodal involvement (n=34) and metastases (n = 44) against 35 healthy men, and 35 patients with benign prostatic hyperplasia, and reported that 7 of the 10 markers studied were significantly increased in PCa patients with bone metastasis [118]. This suggests that PCa DTCs can induce co-opt niches within the bone marrow, leading to both osteoblastic (bone forming) and osteolytic (bone resorption) lesions. The interaction of PCa cells and the bone microenvironment therefore represents a potential novel therapeutic target [119]. Other studies have suggested that the interactions between metastasising PCa cells and bone microenvironment are important for cells to gain survival advantages [120]. In this model, the PCa DTC within the bone micro-environment initiates a cooperative cycle between host osteoclasts and osteoblasts, and leads to cancer cell growth. In this context, it has been shown that PCa promotes osteoblastic lesions that *via* endothelin 1 (ET-1) signaling, which stimulates osteoblasts *via* the E_A_R. Pharmacological targeting of ET-1 signaling has shown considerable promise in mouse models of breast cancer, resulting in decreased tumor burden and metastatic lesions [121].

Moreover, Clines *et al*. have analysed murine primary osteoblast culture to validate ET-1 targets by RT-PCR, and found that ET-1 signaling lead to downregulation of WNT signaling pathway inhibitor, dickkopf homologue 1 (DKK-1) through unknown mechanisms. WNT signaling pathway, which is believed to be the key osteoblast-regulating pathway responsible for normal osteoblast differentiation, and function was upregulated [122]. Similarly, upregulation of DKK-1 has been shown to reverse the effect and lead to osteolytic lesions. This has been demonstrated by Hall *et al*., who transfected osteolytic PC-3 cells with short hairpin RNA (shRNA) to induce osteoblastic activity in murine stromal cells. Additionally, they were able to induce osteolytic phenotype in mixed osteolytic/osteoblastic PCa cell line C4-2B that were transfected with DKK-1 expression vector and injected into tibiae of mice [123]. Nelson *et al*. have reported high ET-1 levels in plasma of patients with metastatic PCa [124]. Taken together, these studies showed that bone microenvironment, and more specifically stem cell bone marrow niche may provide survival advantages for DTCs. However, up to date, this particular field has not yet been explored in more detail. 

## NOVEL THERAPIES

### PCa Targeted Therapy

Advances in the understanding of drug resistance mechanisms have pointed towards a number of potential targeted therapies which preferentially affect malignant cells over adjacent normal tissue with the goal of improving clinical efficacy while reducing systemic toxicity [125]. We will discuss PCa targeted therapies which are currently undergoing trials. As previously discussed, CRPC remain dependent on AR-associated signaling, despite failure of ADT. Various novel agents targeting androgen synthesis or the AR protein itself are currently in use or undergoing clinical development. The most prominent drugs in this category include but are not limited to CYP17A inhibitor, abiraterone acetate (AA), and anti-androgen compounds MDV3100 and ARN-509 [45, 46].

The CYP17A (17α-hydroxylase/C17,20-lyase) enzyme is required for androgen synthesis. Abiraterone acetate (AA) is a potent pregnenolone derivative that irreversibly binds to CYP17A inhibiting its activity, and therefore decreases androgen synthesis in testes, adrenal gland and prostate tumor. AA has been demonstrated to reduce serum androgen levels more effectively than ketoconazole in a phase I clinical trial [126]. Moreover, a phase II clinical trial in men with CRPC that were previously treated with docetaxel has also shown promising results when treated with AA. The phase II clinical trial enrolled fifty eight patients with metastatic CRPC who had failed docetaxel therapy. The primary outcome was a ≥ 50% PSA decline, with objective response by Response Evaluation Criteria in Solid Tumors (RECIST) criteria, changes in Eastern Cooperative Oncology Group (ECOG) performance status (PS), and circulating tumor cell (CTC) number. A 50% or higher decline in PSA was observed in 36% of the patients that received 1,000 mg daily dose of AA with 5 mg twice daily dose of prednisone, with a median time to biochemical progression of 169 days (95% CI, 82 to 200 days). CTC responses to treatment were noted in 34% (10 out of 29) patients [127]. Two phase III clinical trials of AA in docetaxel-treated and docetaxel-naïve patients with CRPC have been completed and are estimated to be concluded in December, 2012, and February 2014 respectively (ClinicalTrials.gov Identifier: NCT00638690, NCT0088-7198) [128]. One phase III clinical trial of 1195 CRPC patients who have previously received docetaxel was recently reported. In the study, the patients were randomly assigned in ratio of 2:1 to receive 5 mg of prednisone twice daily, with either 1000 mg of AA (n=797) or placebo (n=398). With primary endpoint as OS, the study has reported a 3.9 months survival advantage of the AA treatment group compared to the placebo group (hazard ratio, 0.65; 95% confidence interval, 0.54 to 0.77; p < 0.001). Moreover, all secondary end points including PSA progression, progression-free survival (PFS), and PSA response rate favoured the AA treatment group as well. The study has concluded that AA treatment significantly prolonged the OS of CRPC patients [129]. AA has been approved for clinical use in the US and Europe.

Another promising antiandrogen is TAK-700 (trade name Orteronel), an oral, selective, non-steroidal androgen synthesis inhibitor of one of the two enzymatic reactions catalysed by CYP17A1. The drug has been shown to be well tolerated in phase I/II clinical trial on chemotherapy-naïve CRPC patients [130]. Currently, two randomized multicenter phase III clinical trials aiming to investigate the efficacy and safety of TAK-700 in combination with prednisone versus placebo plus prednisone in chemotherapy-naïve and docetaxel-pretreated metastatic CRPC patients is ongoing, and are planned to conclude in 2013 and 2014 respectively (ClinicalTrials.gov Identifier: NCT01193257, NCT01193244) [128].

### Targeting Proliferation and Survival Pathways

A number of candidate drugs targeting pathways that cross-talk with AR-dependent pathways are being evaluated. Phase II clinical trials for the EGFR-targeting compounds gefitinib and erlotinib did not show any substantial activity in CRPC [131, 132]. Similarly, cetuximab, a monoclonal antibody against EGFR, has recently been evaluated in phase II clinical trials in combination with mitoxantrone plus prednisone after docetaxel treatment in metastatic CRPC [133]. The study enrolled a total of 115 patients that failed to respond to docetaxel treatment and demonstrated further metastatic CRPC progression. The participants were divided into two groups: first group received combination therapy of mitoxantrone and prednisone (n = 40), while the second group received the same combination plus cetuximab (n=75). While median time to progression (TTP) and median survival was longer in mitoxantrone/prednisone/cetuximab group (6.6 versus 4.6 months, and 15.7 versus 11.9 months respectively), measurable disease response rate, and PSA response did not show any significant differences. Key grade 3-4 toxicities were generally higher in mitoxantrone /prednisone/cetuximab group. The study concluded that treatment with cetuximab in combination with mitoxantrone and prednisone is not recommended in docetaxel-treated metastatic CRPC [133]. Two phase II clinical trials for A monoclonal antibody against IGFR has been completed and are undergoing evaluation (ClinicalTrials.gov Identifier: NCT00313781) and (ClinicalTrials.gov Identifier: NCT00520481) [128]. A randomized phase II clinical trial was conducted to compare the efficacy of CNTO-328, a monoclonal antibody against IL-6 in combination with mitoxantrone in CRPC patients who have been previously treated with docetaxel. Enrolment of the trials was terminated after interim analysis that revealed more deaths occurring in CNTO-328/mitoxantrone arm (9 versus 4 deaths). The majority of deaths were due to disease progression, with median PFS being higher in mitoxantrone group (228 versus 97 days) [134].

Candidate drugs targeting key survival pathways are also being investigated. In this group, oblimersen, an antisense oligonucleotide targeting the *Bcl-2* inhibitor of apoptosis mRNA has failed to show better activity in combination with docetaxel when compared to docetaxel alone in CRPC patients. A phase II clinical trial enrolled 111 patients, with first arm receiving 75 mg/m^2^ of docetaxel on day 1 (n = 57), and second arm receiving 7 mg/kg/day infusions of oblimersen with same dose of docetaxel on day 5 (n = 54). The treatments were performed for 3 consecutive weeks. A PSA decline of ≥ 30% was observed in fewer patients treated with combination therapy (37% versus 46%). Major toxicity events were reported in 22.8% and 40.7% of patients treated with docetaxel or docetaxel plus oblimersen respectively. The study has reported that the primary end points were not met [135]. Moreover, a randomized, double-blind phase II trial of docetaxel plus prednisone combined with an agent against multiple Bcl-2 family proteins (AT-101) has also been completed, and evaluated (ClinicalTrials.gov Identifier: NCT00571675) [128]. The phase II clinical trial enrolled 221 patients with metastatic and progressing CRPC. The patients were divided into two treatment arms and received 75 mg/m^2^ of docetaxel on day 1, and 5 mg of prednisone orally twice daily every 21 days, with either 40 mg of AT-101 or placebo twice daily orally on days 1 through 3. Despite previously demonstrated activity, treatment with AT-101 did not extend OS when combined with docetaxel and prednisone, and therefore did not meet primary end point. Nevertheless, additional analysis has revealed a potential benefit in high-risk patient group [136].

Meanwhile, an antisense oligonucleotide (custirsen/ OGX011) which targets mRNA of the survival factor clusterin, has demonstrated promising effects in phase II clinical trial for combination therapy with custirsen and docetaxel or mitoxantrone in CRPC patients. Custirsen has been demonstrated to improve the response of taxane-resistant cell lines to chemotherapy in preclinical studies. As such, the phase II clinical trial included patients that showed progressive disease, and were currently receiving or were within 6 months of discontinuing first-line docetaxel treatment. The trial compared docetaxel, prednisone plus custirsen, with mitoxantrone, prednisone plus custirsen. Both custirsen combination regimens were well tolerated, and demonstrated significant PSA response, with ≥50%r PSA decrease in 40% versus 27% in docetaxel/prednisone /custirsen and mitoxantrone /prednisone/custirsen treatment arms respectively [137]. Currently two phase III clinical trials are ongoing which aim to evaluate the effects of custirsen in CRPC patients. One trial will consist of approximately 1000 patients and aims to confirm that adding custirsen to standard docetaxel/prednisone treatment can slow cancer progression, and enhance survival outcome (ClinicalTrials.gov Identifier: NCT01188187) [128]. The second phase III trial will consist of 292 patients, and aims to evaluate the pain palliation benefit of adding custirsen to a taxane-based therapy as second-line chemotherapy in CRPC patients (ClinicalTrials.gov Identifier: NCT01083615) [128].

### Targeting Angiogenesis

As mentioned earlier, vascularisation in tumerous tissue is disorganized, has lower than normal blood flow, and therefore contributes to impaired drug supply to the tumor cells. It has been demonstrated that blocking of VEGFR may lead to a transient restoration of functional blood vessels in a variety of tumors. Consequently, oxygen supply and drug delivery to the tumor cells may be improved [138]. Concomitant with the finding, antiangiogenic agents have been developed, and are currently being evaluated in clinical trials. This category includes agents such as bevacizumab, thalidomide, and sunitinib. Bevacizumab and thalidomide have demonstrated positive activity when used in combination with docetaxel in PC-3 mouse xenograft models and phase II clinical trials [139]. Results from the mouse model indicated that combination therapy of docetaxel, bevacizumab and thalidomide inhibited tumor growth most effectively. In parallel, patients with progressive CRPC received intravenous docetaxel and bevacizumab plus oral thalidomide and prednisone with the primary end point being ≥50% PSA decrease and secondary end point was time to progression (TTP), OS, and safety. Overall, 90% of the patients had ≥50% PSA decline, with 88% showing a 30% or larger decline within the first 3 months of therapy. Median TTP was 18.3 months, and OS was 28.2 months. The study has thus shown that bevacizumab, thalidomide, and docetaxel combination therapy is highly active, and encouraging, given the generally poor prognosis of CRPC patients with metastatic disease [139]. A second phase II clinical trial evaluated thalidomide and docetaxel combination therapy versus docetaxel alone. The study enrolled 75 patients with CRPC. The first group received 30 mg/m^2^ intravenous docetaxel injections for 3 consecutive weeks (n=25), while the second group received docetaxel at the same dose and schedule, plus 200 mg thalidomide orally each day (n=50). Serum PSA levels, and radiographic scans were used to determine PSA decline and TTP. After a median follow-up time of 26.4 months, proportion of patients with 50% or larger PSA decline was greater in docetaxel/thalidomide group (53% versus 37%). Median progression-free survival was not statistically significantly different, although the median OS was greater in docetaxel/thalidomide group (68.2% versus 42.9%). The study has concluded that thalidomide used in combination with docetaxel leads to encouraging PSA decline and overall median survival [140]. While these phase II clinical trials using bevacizumab in combination with docetaxel and thalidomide showed promising results, a recent phase III clinical trial of bevacizumab failed to show a survival benefit in combination with docetaxel and prednisone compared to docetaxel and prednisone alone (ClinicalTrials.gov Identifier: NCT00110214) [128]. Additional phase III clinical trial of a more potent angiogenesis inhibitor, aflibercept, in metastatic CRPC has been completed recently. The study is currently undergoing evaluation, and the final results are planned to be published in June, 2012 (ClinicalTrials.gov Identifier: NCT00519285) [128]. Phase III clinical trial for a multitarget inhibitor of VEGF and platelet derived growth factor receptor (PDGFR), sunitinib, was discontinued at the second interim analysis on September 2010 by the data monitoring committee due to disease progression, adverse events, and consent withdrawal in both the treatment arms. While the study was able to demonstrate improvement in median PFS in metastatic CRPC patient group treated with prednisone and sunitinib combination therapy versus prednisone plus placebo group, OS between the two treatment groups did not show significant differences [141].

### Targeting Microenvironment for Treatment of Distant and Bone Metastasis

Since it is now appreciated that microenvironment plays an important role in metastatic PCa progression, a number of candidate therapies have been developed to target the interaction between DTC and their microenvironment. However, targeting only the microenvironment may not be an effective approach, as exemplified by the failure of a selective E_A_R antagonist, atrasentan. Atrasentan did not reduce the risk of disease progression relative to placebo group in a multinational, double-blind, placebo-controlled phase III trials of 809 patients with metastatic CRPC [142]. A phase III clinical trial with approximately 930 patients with CRPC is undergoing evaluation of combination therapy with docetaxel and atrasentan versus docetaxel and placebo, and is estimated to reach the primary objective on March, 2014 (ClinicalTrials.gov Identifier: NCT00134056) [128]. Another E_A_R antagonist, zibotentan has recently been reported to fail at phase 3 clinical trial (ClinicalTrials.gov Identifier: NCT00617669) [128, 143].

The use of radioisotopes and radiolabelled monoclonal antibodies that target both metastatic PCa and microenvironment cells has shown some promising results [144]. In a randomized phase II clinical trial with 103 patients with CRPC that were receiving ketoconazole and doxorubicin, or estramustine and vinblastine chemotherapy, after induction chemotherapy was complete, 72 patients were randomly assigned to receive consolidation therapy with doxorubicin, and with or without β-emitting strontium-89 (^89^SR). The study has reported a significant improvement in OS (27.7 months versus 16.6 months, P = 0.001) in favour of chemotherapy followed by consolidation when compared to chemotherapy alone [145]. A confirmatory phase III clinical trial is currently ongoing, and will conclude in October, 2014 (ClinicalTrials.gov Identifier: NCT00024167) [128]. Moreover, similar results have been reported from phase II evaluating combination therapy with β-emitting samarium-153 (^153^Sm) and docetaxel [146], and α-emitting radium-223 (^223^Ra; alpharadin) as monotherapy [147], with promising results. A phase II clinical trial for antibody against prostate-specific membrane antigen (PSMA), conjugated with β-emitting lutetium-177 (^177^Lu) is currently recruiting patients with metastatic CRPC (ClinicalTrials.gov Identifier: NCT00195039)[128].

Additionally, dasatinib, an Src/SFK inhibitor that has already been approved for treatment of some forms of leukaemia, has recently concluded phase II trials on chemotherapy-naïve CRPC patients. In the study, 48 patients received daily oral dose of 100 mg dasatinib. The primary measure in this group was a composite lack of disease progression with accordance to Prostate Cancer Working Group 2 criteria that was determined every 12 weeks during the study period. Lack of disease progression was observed in 44% of the patients by week 12 and 17% of the patients by week 24, and provided evidence for promising dasatinib activity in bone (ClinicalTrials.gov Identifier: NCT00385580) [128, 148]. Another promising phase II clinical trial for microenvironment-targeting drug, a monoclonal antibody against the chemokine CCL2, CNTO-888 (Centocor, Horsham, PA, USA) has been completed, and is currently undergoing evaluation (ClinicalTrials.gov Identifier: NCT00992186) [128].

### Immunotherapy as the Effective Approach for Targeting Distant and Bone Metastasis

Recent development of various immunotherapies designed to target PCa-specific antigens has shed new light in treating advanced PCa as well. Most prominent therapy in this category is sipuleuceal-T (trade name Provenge) that has been approved by Food and Drug Administration (FDA) for treating asymptomatic or minimally symptomatic metastatic CRPC on 29^th^ of April, in 2010 [149]. Sipuleucel-T is a dendritic cell (DC)-based immunotherapy that is designed to stimulate immune response to prostatic acid phosphatase (PAP) which has been demonstrated to be specific for prostate tissue and most of prostate carcinomas [150]. The approval of sipuleucel-T followed the most recent randomized multicenter phase III clinical trial that enrolled 512 patients that were randomly assigned in a 2:1 ration to receive either sipuleucel-T or placebo every 2 weeks for a total of three infusions. OS was set as the primary end point, and time to objective disease progression was the secondary end point of the study. The OS was defined as the time from randomization until the death of the patient from any cause. Objective disease progression was determined by radiographic studies, with progression defined as 50% increase in sum of index lesion diameters, new appearance or unequivocal progression of nonindex lesions, at least two new lesions on bone scanning, or new pathologic fractures or spinal cord compressions. A total of 92.2% of patients in sipuleuceal-T has completed the trial. Statistical analysis of the data has revealed that sipuleucel-T therapy has reduced the risk of death by 22% and extended the median survival by 4.1 months (25.8 months OS) compared to placebo (21.7 months OS). Herewith, sipuleucel-T has increased the probability of survival by 8.7% compared to placebo. No substantial differences in time to objective disease progression between sipuleucel-T and placebo treatment groups were observed. Adverse events were more frequently observed in sipuleucel-T treatment group, including chills, fever, and headaches. Consequently, the study has concluded that sipuleucel-T has prolonged OS among men with metastatic CRPC (ClinicalTrials.gov Identifier NCT00065442) [128, 151].

While Sipuleucel-T is the first and only immunotherapy that has been demonstrate to be effective in CRPC patients in phase III clinical trials, other immunotherapies may also be on the way. In this context, blocking of negative immune regulator; cytotoxic T lymphocyte antigen 4 (CTLA-4) has been demonstrated to augment and prolong CD4 and/or CD8 T-cell activity, leading to immune response and increased antitumor activity. Firstly, a pilot study performed by Small and colleagues have concluded that blocking of CTLA-4 with human anti-CTLA-4 antibody; ipilimumab in patients with CRPC has PSA-modulating effects, is well-tolerated, and does not lead to significant clinical autoimmunity [152]. Similar findings have been demonstrated in a dose-escalation phase I clinical trial of poxviral-based vaccine targeting PSA; PSA-Tricom and the CTLA-4 antibody; ipilimumab. The study enrolled 30 patients with metastatic CRPC. The patients received 2x10(8) plaque-forming units of PSA-Tricom subcutaneously on day 1, and subsequent monthly boosts of 1x10(9) units of PSA-Tricom starting on day 15. Intravenous injections of ipilimumab were performed monthly, starting at day 15, in doses of 1, 3, 5, and 10 mg/kg. The study has reported no dose-limiting toxicity effects. Grade 1 and 2 vaccination-site reactions were the most common toxic effects. Most patients had grade 2 immune-related adverse events (n=21), and grade 3 or 4 immune-related events such as diarrhea or colitis, rash, increased aminotransferases, endocrine immune-related adverse events, and neutropenia were uncommon. Only 1 out of 6 patients that were previously treated with chemotherapy showed a PSA decline from baseline. In chemotherapy-naïve cohort, 24 had PSA decline from baseline, with 6 of the patients showing a decline greater than 50% (ClinicalTrials.gov Identifier NCT00113984) [128, 153]. Moreover, Yu P and colleagues have used an established murine transgenic adenocarcinoma of mouse prostate (TRAMP)-C2 prostate tumor model to investigate the effects of simultaneous inhibition of negative immune regulators; CTLA-4 and programmed death ligand 1 (PD-L1), and immune stimulation with IL-15. The study has reported that blocking the two negative immune regulators and stimulating the immune response with IL-15 has lead to enhanced immune response and increased antitumor activity [154]. Multiple phase 2 and 3 clinical trials are currently ongoing to investigate the benefits of ipilimumab in combination with other therapies [155].

## CONCLUSION

Although the underlying mechanisms of the PCa drug resistance are being elucidated and there are improvements in advanced PCa treatments, a stubbornly large percentage of PCa patients continue to progress through lethal metastatic disease. The absolute numbers of men affected by metastatic PCa are likely to increase significantly in the next decade reflecting demographics. Thus, advanced PCa will continue to represent a major clinical challenge and a significant burden on the healthcare system and society. Current treatments for metastatic disease centre around ADT, and are aimed at delaying disease progression. While these are initially effective in the majority of the cases, many PCa cases will eventually fail to respond to ADT and become CRPC. As we have outlined here, currently there are limited therapeutic options available for CRPC, although continued developments have increased the number of agents now available to patients. Importantly, however, no expectation of cure is yet available for CRPC. We have outlined the diverse mechanism of treatment resistance which have been identified in PCa. We have also described current efforts to develop new therapeutic combinations being tested in CRPC. Indeed the last decade has seen several exciting developments including the approval of new drugs which target androgen biosynthesis or elicit PCa-specific immune response, and which hold much clinical promise. As our understandings of the underlying molecular and cellular mechanisms of PCa are improving, we hope that additional therapeutic opportunities for PCa will soon become available.

## Figures and Tables

**Fig. (1):**
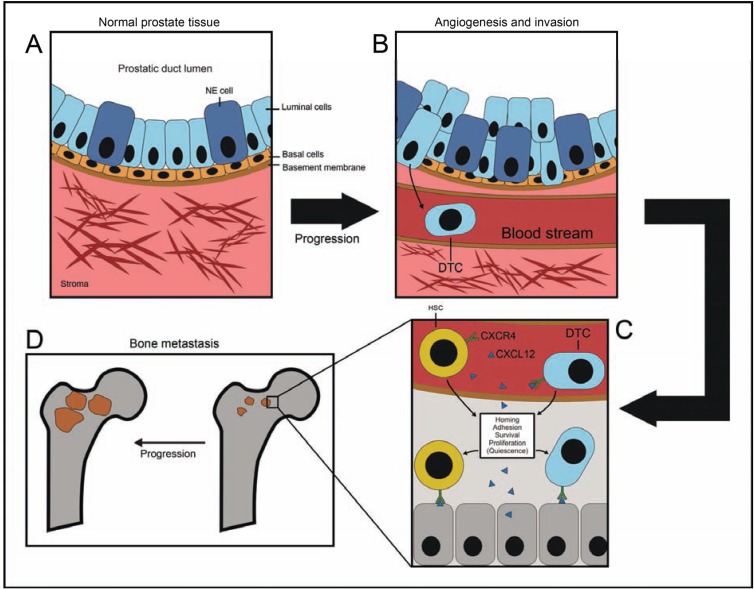
PCa progression. **A**; normal prostate tissue with intact basement membrane, and organized luminal and neuroendocrine (NE) cells, **B**; PCa progression is signified by disorganized expansion of luminal and NE cells, angiogenesis, and increased invasiveness of PCa cells, **C**; chemokine rich milieu of the bone marrow combined with adhesion molecules expressed within HSC niche play an important role in recruiting DTCs, **D**; extravasation of PCa into bone microenvironment, and bone metastasis.

**Fig. (2):**
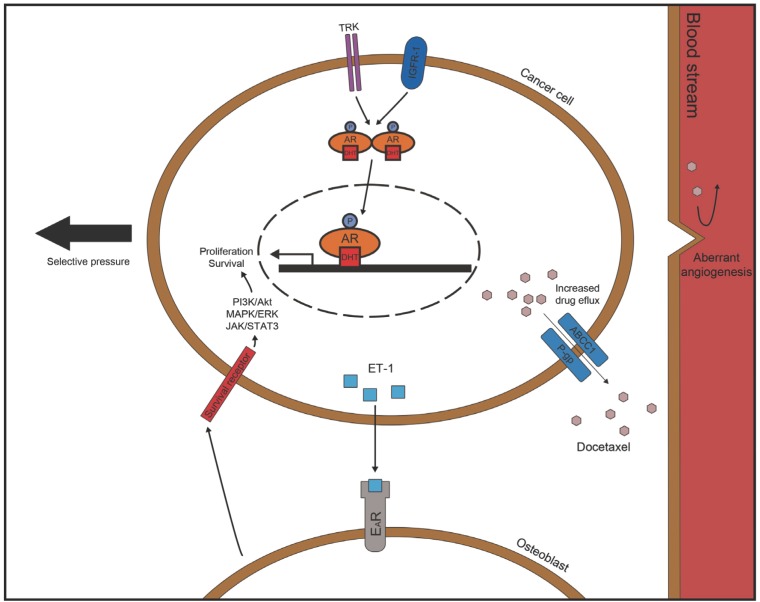
PCa drug resistance may involve both intrinsic and extrinsic mechanism that include activation of AR receptor, activation of alternative survival pathway such as Akt/PI3K, MAPK/ERK or JAK/STAT3, cross talk of between cancer cells and surround microenvironment, and selective pressure due to various therapies. Additionally, more general drug resistance mechanisms may involve expression of efflux pump, and aberrant angiogenesis.

**Fig. (3):**
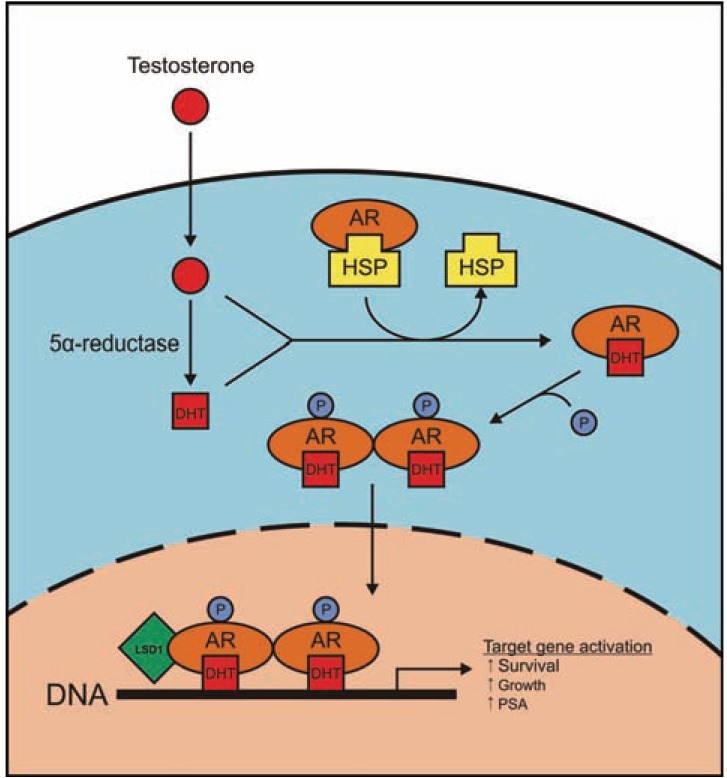
Inside a cell, testosterone is converted into DHT by 5α-reductase. In presence of either testosterone or DHT, AR is released from heat shock proteins (HSP), and gets phosphorylated and transported into nucleus as a homo-dimer. Once inside the nucleus, AR homo-dimer is joined with various coregulators such as LSD1, which leads to transcription activation, and consequently increased survival and growth of the cell.

**Table 1.** Novel Targeted Therapies for Treatment of Advanced PCa

Drug	Targeted PCa Stage	Mode of Action	Developmental Stage	Common Adverse Events
Cabazitaxel	Docetaxel-resistant CRPC	Microtubule inhibitor	Approved by FDA on June 17, 2010	Leucopenia, anaemia, fatigue and asthenia, with grade 3 or higher events: neutropenia, diarrhea and febrile neutropenia
Abiraterone	Metastatic CRPC	CYP17A1 inhibitor	Approved by FDA on April 28, 2011	Joint swelling or discomfort, low levels of potassium in the blood, fluid retention, muscle discomfort, hot flashes, diarrhea, urinary tract infection, cough, high blood pressure, heartbeat disorders, urinary frequency, increased night-time urination, upset stomach or indigestion and upper respiratory tract infection
TAK-700	Metastatic CRPC	CYP17A1 inhibitor	Ongoing phase III clinical trial	Fatigue, nausea and constipation, with grade 3 or higher events: fatigue and diarrhea
Custirsen	CRPC	Survival factor, clusterin synthesis inhibitor	Ongoing two phase III clinical trials	Fatigue, nausea, diarrhea, anorexia, chills, vomiting, fever, taste disturbance, cough, neuropathy, joint pain, peripheral edema, hair loss, back pain and constipation
^89^SR	CRPC with bone metastasis	Bone-homing radioisotope, bone-targeted radiation therapy	Ongoing confirmatory phase III clinical trial	Neutropenia, dyspepsia, oesophagitis, gastritis, oedema, fatigue, deep venous thrombosis
^223^Ra	CRPC with bone metastasis	Bone-homing radioisotope, bone-targeted radiation therapy	Ongoing phase III clinical trial	Mild transient bone marrow toxicity
Dasatinib	CRPC	Src family tyrosine kinsae inhibitor	Ongoing phase III clinical trial	Fatigue, nausea, diarrhea, headache, and anorexia
Sipuleucel-T	Asymptomatic or minimally symptomatic metastatic CRPC	Active immunization against PAP, DC-based immunotherapy	Approved by FDA on April 29, 2010	Chills, fatigue, back pain, pyrexia, nausia, arthralgia, citrate toxicity, vomiting, headache, anemia, limb pain, dizziness, paresthesia, constipation, musculoskeletal pain, pain, oral paresthesia, asthenia, diarrhea

## ABBREVIATIONS

ADT: = Androgen Deprivation Therapy
DTCs: = Disseminated Tumor Cells
MP: = Metastatic Progression
HSCs: = Hematopoietic Stem Cells
CRPC: = Castration Resistant Prostate Cancer
OS: = Overall Survival
EMDR: = Environment-Mediated Drug Resistance
TTP: = Time to Progression
